# Adoption of direct-acting antiviral medications for hepatitis C: a retrospective observational study

**DOI:** 10.1186/s12913-019-4349-x

**Published:** 2019-07-25

**Authors:** Leah L. Zullig, Haresh L. Bhatia, Ziad F. Gellad, Mark Eatherly, Rochelle Henderson, Hayden B. Bosworth

**Affiliations:** 1 Center of Innovation to Accelerate Discovery and Practice Transformation (ADAPT), Durham Veterans Affairs Health Care Center, 411 West Chapel Hill Street, Suite 600, Durham, NC 27701 USA; 2 Department of Population Health Sciences, Duke University Medical Center, Durham, NC USA; 3 Express Scripts Holding Company, St. Louis, MO USA; 4 Division of Gastroenterology, Duke University Medical Center, Durham, NC USA; 5 Department of Psychiatry and Behavioral Sciences, Duke University Medical Center, Durham, NC USA

## Abstract

**Background:**

Approximately 3.5 million Americans are infected with the hepatitis C virus (HCV). Although many patients with HCV are asymptomatic, HCV is the leading cause of infection-related death in the U.S. With advances in curative medication therapy for HCV, many of these deaths are preventable. Access to innovative therapies may be unevenly distributed. Our objective was to describe medication prescribers’ adoption of innovative HCV pharmacotherapy across prescriber, geographical location, and time.

**Methods:**

This is a retrospective, secondary data analysis among a national cohort of patients prescribed direct-acting antiviral HCV medications with curative intent. We assessed prescriptions by time, geographic location, and provider type.

**Results:**

The peak of the adoption rate occurred within 45 days; nearly one-sixth of all prescribers had already prescribed one of the new drugs. Geographical regions (Midwest, South, and West all *p* ≥ 0.05) nor gender (*p* = 0.455) of a prescriber impacted adoption. Similarly, patient income did not influence the likelihood of a prescriber to adopt the new drugs earlier (*p* = 0.175). Gastroenterologists or hepatologists were more likely earlier adopters compared to primary care physicians (*p* = 0.01).

**Conclusions:**

Because of the relative advantage of newer therapies, we anticipated that there would be an initial surge as early adopters prescribed the new medications and use would dwindle over time as the initial HCV cohort was cured. The data demonstrate that our hypothesis is essentially supported. There is a reduction in prescriptions at approximately 5 months post-approval and treatment is typically required for 3 months. There has been a surge in clinicians’ adoption of innovative HCV treatments. As patients are cured of their infection, we anticipate a decreased need for chronic management of HCV.

**Trial registration:**

Not applicable.

**Keywords:**

Dissemination researchHealth services researchInfectious diseaseHepatitis C

## Background

An estimated 3.5 million Americans are living with the Hepatitis C virus (HCV) and many are unaware of their infection [1, 2]. Although most patients do not exhibit symptoms, without treatment, approximately 20–30% of patients will develop cirrhosis and resulting complications over the next 2 to 3 decades [3]. According to the Centers for Disease Control and Prevention, HCV is responsible for more American deaths than any other infectious disease [4]. Furthermore, while the prevalence of the disease is decreasing in the United States, the incidence of advanced liver disease associated with chronic HCV infection is predicted to increase, resulting in an estimated peak societal cost of 9.1 billion in 2024 [5, 6].

Historically, the standard treatment for patients with chronic HCV was a dual combination regimen of pegylated interferon and ribavirin [7]. Most patients required therapy for 48 weeks and experienced significant side effects including fatigue, anemia, and depression which were barriers to completion of treatment [8]. Cure rates were moderate, approximately 46–82% depending on genotype [9, 10]. In 2011, two direct-acting antivirals were approved by the FDA, boceprevir and telaprevir, for the treatment of patient with genotype 1 HCV. Adoption of these medications was limited by the need to use them in combination with existing, poorly tolerated therapies. Several other medications entered the market in 2013 including the first once daily protease inhibitor, simeprevir, and the first polymerase inhibitor, sofosbuvir. While these two medications were used together off-label to treat genotype 1 infection without interferon, it was not until October 2014 when the FDA approved the first all-oral single combination pill treat genotype 1 HCV, ledipasvir/sofosbuvir. In clinical trials, 12 weeks of treatment with ledipasvir/sofosbuvir was associated with a 94% cure rate for previously treated patients and a 97% cure rate in previously untreated patients [11]. Since its initial approval, ledipasvir/sofosbuvir has been approved for patients with different genotypes and can be used to treat patients with HCV-related complications including cirrhosis or liver transplant recipients [12, 13]. In December 2014, another interferon-free regimen, ombitasvir/paritaprevir/ritonavir, was approved; ombitasvir/paritaprevir/ritonavir trials demonstrated similarly high cure rate for patients with genotype 1 HCV [14]. In addition to their improvement in cure rate, direct-acting antivirals were better tolerated than interferon by patients because of the significantly reduced side effects and shorter duration of treatment.

There is often an imbalance in the introduction and diffusion of new treatments across the United States. This imbalance in access to new treatments may be associated with geographic and/or socioeconomic status. Given the recent introduction of direct-acting antiviral treatment for HCV, little is known about the pattern of diffusion of these medications. Also unknown is the source of these variations and whether these variations are impacted by characteristics of the prescribers. From a consumer perspective, there is evidence that wealthy patients and consumers drive the adoption of emerging technologies in areas of higher income inequality [15]. In health care, income inequality is associated with greater use of specialty care, and this demand may be driven by a preference among higher income health care consumers for more “cutting-edge” care [16]. Therefore, areas with a higher proportion of wealthy people may experience increased demand for cutting-edge medical technologies such as HCV treatment.

From the perspective of prescribers or providers, we theorized that the effect of income inequality on adoption of HCV medications is mediated by health care market factors. Over time the physician distribution in the United States has become increasingly unequal. Specialists are concentrated in geographic areas where there is a higher penetration of well-off, well-insured health care consumers [17, 18]. Economic theory suggests that the clustering of specialty care may also spur innovation and knowledge diffusion [19]. As as result, these already more affluent areas may foster dense specialist markets where prescribers may have more resources and interest to maintain greater awareness of and inclination to adopt emerging medical technologies such as use of HCV medications.

If there is variation in the adoption of innovative HCV medications, these differences in the marketplace may continue the described healthcare disparities in the US healthcare system. We have not identified studies that explored whether area-level income inequality, net of individual income, has any bearing on the diffusion of emerging medical technologies in the context of HCV. It is also not known whether patients receiving care in different practice settings or geographic areas have differential access to cutting edge treatment for HCV. Our objective is to evaluate whether there is regional and socioeconomic variation or differences by provider type in the adoption (or uptake) of new HCV medications.

## Methods

### Research design

This is a retrospective, observational claims analysis using prescription claims data from a national pharmacy benefits management company. Data were obtained for prescriptions from October 1, 2014 through June 30, 2016, spanning 18 months following the respective release dates of ledipasvir/sofosbuvir (October 1, 2014) and ombitasvir/paritaprevir/ritonavir (January 1, 2015). We used the date that the first prescription was filled as a proxy measure for the date that the first prescription was written by their health care provider. The date of the first prescription fill was used as the index date.

### Study population

Inclusion was limited to medical practitioners (i.e., prescribers) who wrote at least one prescription each for HCV medications ledipasvir/sofosbuvir and ombitasvir/paritaprevir/ritonavir during the study period. Prescribers were excluded from analysis if they were missing a National Provider Index (NPI) number, were in an invalid state (e.g., U.S. Armed Forces stationed in Europe), were prescribing for patient younger than 18 years of age, or were practicing in an area with missing or 0 median income data.

### Theoretical model

**Fig. 1:**
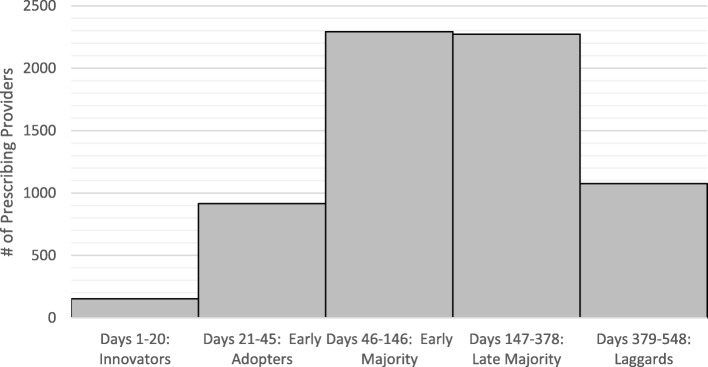
Prescriber categorization by first Rx day

The Diffusion of Innovation Theory is a social science theory that was developed to explain how an innovative product or idea diffuses, or spreads, through a specific population over time [20]. The end result of the diffusion is that members of the population, in our case, clinical prescribers, adopt a new behavior. One key construct of the Diffusion of Innovation Theory is that the prescribers must perceive the product (i.e., HCV medications) to be new and innovative. Rogers’ theory also asserts that adoption of an innovative product is a process; it does not occur simultaneously. There are five established categories of adopters: innovators (i.e., first to try the new product), early adopters (i.e. opinion leaders), early majority (i.e., not leaders, but adopt a product before the average prescriber), late majority (i.e., prescribers who will only try a product after it has been tried by the majority), and laggards (i.e., prescribers who are skeptical of change). We classified HCV medication prescribers into these five categories based on when the first prescription was written and filled and anticipated that the majority of prescribers would fall in the middle two categories (e.g., early and late majority). These prescriber categories were defined based on the proportion consistent with Rogers’ theoretical model – innovators (2.28%), early adaptors (13.63%), early majority (34.18%), late majority (33.86%), and laggards (16.05%), consistent with the theoretical model (Fig. 1).

### Outcome measure

The primary outcome measure was the adoption, or timeliness of uptake, of the new HCV medications by prescribers. To address the issue of different release dates for ledipasvir/sofosbuvir and ombitasvir/paritaprevir/ritonavir, a ‘*first Rx day*’ was created as the ‘*fill-date minus the respective release date*’ for either of the drugs. The *first Rx day* served as a representative of the first prescription. For example, the *first Rx day* for NPI 12345 would be 12 if her first HCV medication prescription is on 10/12/2014 and happens to be for ledipasvir/sofosbuvir (released on 10/01/2014); similarly, the *first Rx day* for NPI 56789 would be 2 if her first HCV medication prescription is on 01/02/2015 and happens to be for ombitasvir/paritaprevir/ritonavir (released on 01/01/2015).

### Covariates

Region was created as a categorical variable and was assigned to the prescribers based on the state associated with their respective primary practice address. We used the U.S. Census Bureau’s categorizations to assign states into regions [21]. Median income at the zip code level data were used as proxy for income inequality. We identified median income for individual NPIs by matching zip codes of their primary practice address. The median zip code level median income data were obtained from 2016 Esri business analyst database (using Esri ArcMap v 10.3.1) [22]. The estimates of 2016 demographic data were estimated with the 2016 census data [23]. The median income at zip-code level thus identified for respective NPIs was modified to represent a multiple of 10,000 (e.g., median zip code level income value of 2.59 was used for the corresponding value of $25,900 indicated in census data).

We also considered other prescriber characteristics including sex and specialization. For this analysis, a combination of specialization and sub-specialization for individual NPIs was used to categorize them into one of the four distinct groups: gastroenterologists and hepatologists, infectious disease specialists, other specialists, and primary care (e.g., including family and internal medicine practitioners). No midlevel providers (i.e., nurse practitioners or physician assistants) were listed as infectious disease or hepatic care providers. Thus, all midlevel providers were considered primary care providers.

### Statistical analysis

We report descriptive statistics and bivariate differences between the outcome groups using ANOVA for continuous variable (median income in a zip-code) and chi-square tests for categorical variables. We performed multivariate ordinal logistic regression analyses to estimate the association of covariates with the odds of uptake (e.g., early adoption, late adoption).

For the multivariate analysis, the outcome of odds of medication uptake, is an ordinal categorical variable; the independent variables sex, region, and specialty were used as categorical variables, while the median income was used as a continuous variable. For the categorical independent variables – Northeast was used as a reference for region, female was used as a reference for sex, and primary care prescriber group was used as a reference for specialization.

All analyses were conducted using ORDINAL package of R [64-bit version 3.4.0 (2017-04-21)].

## Results

### Descriptive findings

We identified 6944 unique prescribers. After limiting our analysis to prescribers with valid information associated with their NPI regarding their sex and zip code information, and who had written a prescription to an adult aged 18 years or older for one of the drugs of interest during the study time period, there were 6712 unique prescribers in the analytic cohort.

The results indicated a wide variation in the *first Rx day* by the respective NPIs, ranging from 2 to 548. The frequency plot of new HCV medication adoption (Fig. 1) indicates a fast adoption as the new medications were introduced. At the peak of the adoption rate, within 45 days, nearly one-sixth of all prescribers had already prescribed one of the new drugs. More than 50% of the prescribers issued their first prescription for one of the drugs within 5 months of their release dates. The percentages indicated are the nearest to the proportions suggested by Rogers’ theory (2.5, 13, 34, 34, and 16% for Innovators, Early Adopters, Early Majority, Late Majority, and Laggards, respectively).

Table 1 lists the baseline characteristics across prescriber adoption categories. Prescribers of the new HCV medications varied in regional locations with 32.2% in the Northeast, 14.08% in the Midwest, 37.57% in the South, and 16.15% in the West. Overall, 28.1% of the prescribers were women. The chi-square analysis for association between geographic regions with the outcome (adoption category) did not indicate that being from a specific geographical location a prescriber would be more likely to be an early prescriber of the new drugs or that the prescriber would be more likely to be characterized into a specific adoption category (*p* = 0.654). However, the chi-square analyses for prescriber sex indicated that female providers tended to be early adopters, influencing the initial hump in the early prescribing of the new drugs. Male prescribers were more likely to be early majority following the normal distribution of the adoption curve (p0.001). The chi-square analyses for specialization category indicated that gastroenterologists and hepatologists are more likely to be early adopters, but some of the primary care providers are more likely to be innovators (*p* <  0.001). The Analysis of Variance (ANOVA) test for income distribution across the five outcome categories indicated that the median income at zip code level did not influence early adoption of the new drugs (*p* = 0.061).

**Table 1:** Baseline characteristics across prescriber adoption categories

Adoption Category	Laggards *n* = 1077(SD)	Late Majority *n* = 2273	Early Majority *n* = 2294	Early Adopters *n* = 915	Innovators *n* = 153
Regions^Δ^ n (%)					
Northeast	327 (15.1%)	721 (33.4%)	756 (35.0%)	308 (14.3%)	49 (2.3%)
Midwest	165 (17.5%)	327 (34.6%)	318 (33.7%)	117 (12.4%)	18 (1.9%)
South	403 (16.0%)	878 (34.8%)	840 (33.3%)	345 (13.7%)	56 (2.2%)
West	182 (16.8%)	347 (32.0%)	380 (35.1%)	145 (13.4%)	30 (2.8%)
Gender* n (%)					
Female	317 (16.8%)	657 (34.8%)	581 (30.8%)	279 (14.8%)	52 (2.8%)
Specialty Category** n (%)					
Primary Care	291 (17.3%)	613 (36.4%)	494 (29.3%)	239 (14.2%)	48 (2.8%)
Regular Specialists (other than Infectious Disease specialists, and Gastroenterologists & Hepatologists)	58 (14.3%)	158 (38.9%)	136 (33.5%)	46 (11.3%)	8 (2.0%)
Infectious Disease Specialists	126 (18.6%)	227 (33.5%)	224 (33.0%)	87 (12.8%)	14 (2.1%)
Gastroenterologists & Hepatologists	602 (15.3%)	1275 (32.3%)	1440 (36.5%)	543 (13.8%)	83 (2.1%)
Median Income (by Zip Code)^Δ^					
Median Income (by Zip Code)	5.83 (2.63)	5.87 (2.69)	5.77 (2.59)	5.99 (2.90)	6.32 (2.95)

^Δ^p - NS; * *p* < 0.05; ** *p* < < 0.01

### Multivariate findings

The results presented in Table 2 indicate the influence of physician specialty on the adoption of new HCV medications. Gastroenterologists or hepatologists were 17.6% more likely to be in an earlier adoption category compared to primary care prescribers (*p* = 0.01). For example, if a male primary care prescriber from a given zip code was categorized in Early Majority, another male prescriber from the same zip code practicing gastroenterology or hepatology would be 18% more likely to be in Early Adopters or Innovators category. However, prescribers in Infectious Disease practice or other Specialties (Pediatrics, Pathology, Hematologists, Oncologists, etc.) did not show an adoption behavior that was different from that of the primary care prescribers (p_infectious_disease_ = 0.885; p_reg.specialty_ = 0.951). Geographical region and sex of a prescriber had little or no influence on the adoption of the new drugs. Thus, neither geographical region (Midwest = 0.055, South = 0.228, West = .598) nor gender (*p* = 0.455) of a prescriber would indicate whether s/he would be quick (or slow) to adopt the new drug or would more likely be in a specific adoption category described in Rogers’ Theory. Similarly, income level did not influence the likelihood of a prescriber to adopt the new drugs quicker (*p* = 0.175).

**Table 2:** β-values (slope parameters) from the ordinal logistic regression

Slope (β) parameter for	β-Estimate (95% CI)	Standard Error	Wald Chi-Square	*P*-Value	Odds Ratio	95% Confidence Limits	
Northeast Region	Reference						
Midwest Region	−0.14 (−0.28, 0.00)	0.0721	3.684	0.055	0.871	0.756	1.003
South Region	− 0.07 (− 0.17, 0.04)	0.0547	1.4505	0.228	0.936	0.841	1.042
West Region	−0.03 (− 0.16, 0.10)	0.0678	0.2778	0.598	0.965	0.845	1.102
Gender – Female	Reference						
Gender – Male	−0.04 (−0.16, 0.07)	0.058	0.5577	0.455	0.958	0.855	1.073
Primary Care	Reference						
Regular Specialty	0.01 (−0.19, 0.22)	0.1059	0.0038	0.951	1.007	0.818	1.239
Infectious Disease*	−0.01 (−0.18, 0.16)	0.0854	0.021	0.885	0.988	0.835	1.168
Gastroenterologists & Hepatologists	0.17 (0.04, 0.29)	0.0623	6.7374	0.009	1.176	1.04	1.328
Median Income	0.01 (−0.01, 0.03)	8.60E-07	1.8362	0.175	1	1	1

*p < 0.05

## Discussion

New HCV medications have several advantages over older treatment regimens: [1] higher cure rate; [2] lower side effect profile; and [3] shorter duration of therapy potentially reducing adherence burden [24]. Because of the relative advantage of newer therapies, we anticipated that there would be an initial surge as early adopters demanded the new medications and use would dwindle over time as the initial HCV cohort was cured. These data demonstrate that our hypothesis was essentially supported. There was a reduction in prescriptions at approximately 5 months post-approval and treatment is typically required for 3 months. This suggests that there may have been an early start up prior to initial approval that was not fully accounted for by our method of analysis.

Our analysis determined that prescribers from the gastroenterology or hepatology community had a higher propensity of early adoption of the new HCV medications, even when compared to infectious disease and other specialties. This finding may suggest that practitioners with specific field of practice related to the Hepatitis C treatment have faster adoption behavior..

There are several factors that may have impacted providers’ use of these medications. There were approximately 90 days between the approval of the two medications. The ombitasvir-combination drug therapy was approved later and was slightly less expensive. Medications may have been included in some formularies, but not others which could have impacted coverage for patients. Initially, Express Scripts included obmitasvir/paritaprevir on their national preferred formulary and excluded ledipasvir/sofosbuvir. Thus, clients that select the national preferred formulary would have to pay out of pocket.

While there is enthusiasm from patients and their providers about the clinical outcomes from these novel therapies, the cost has mitigated enthusiasm from some payers. Despite this, cost-effectiveness analyses have suggested that the long-term pay-off makes the initial investment worth it (high cost up front for drugs, but future cost savings in reduced hospitalizations and fewer liver transplants, etc.) [25].

This analysis has several limitations. We classified all midlevel providers as primary care when, in fact, many midlevel providers specialize in a clinical area. Our analysis does not account for state-level policies that might impact adoption. Some geographic areas data may not be representative of their underlying HCV patient population because Express Scripts has less market penetration is some areas compared to others.

Despite these limitations, our analysis also has several strengths. The analyses include claims from multiple payers including commercial insurers, Medicare, and Medicaid; thus, it covers claims with a variety of coverage policies for novel HCV medications. Additionally, Express Scripts is a large, national company with a sizeable beneficiary population, making this a unique data set with which to assess this research question. Finally, to our knowledge, this is the first study of large-scale adoption of these drugs in the first 18 months following their FDA approval.

In summary, we identified an initial surge as early adopters prescribed the new medications. There was a reduction in prescriptions at approximately 5 months post-approval, which is anticipated since the HCV treatment is typically required for 3 months. Because of the uptake in clinicians’ adoption of innovative and curative HCV we anticipate that there will be a significant reduction in chronic HCV infections, which significantly improve morbidity and mortality related to HCV.

## Conclusions

These data suggest that an initial surge of early adopters prescribed the new medications and use dwindled over time as the initial HCV cohort was cured, including many patients who had been delaying therapy in anticipation of these new drugs. There was a reduction in prescriptions at approximately 5 months post-approval and treatment is typically required for 3 months. There has been a surge in clinicians’ adoption of innovation HCV treatments. As patients are cured of their infection, we anticipate a decreased need for chronic management of HCV. As innovative therapies come to market for HCV and for other conditions, it is important to evaluate prescribing patterns to ensure that appropriate patients have access to cutting-edge treatments regardless of geography or other characteristics.

## Data Availability

The datasets analyzed during the current study are not publicly available because they are proprietary to Express Scripts and contain sensitive patient information, but may available from the corresponding author on reasonable request.

## Abbreviations

ANOVA: Analysis of variance
FDA: Food and Drug Administration
HCV: Hepatitis C virus
NPI: National Provider Identifier
US: United States
